# Decursinol from *Angelica gigas* Nakai enhances endometrial receptivity during implantation

**DOI:** 10.1186/s12906-020-2822-z

**Published:** 2020-02-05

**Authors:** Seong-Eun Kim, Joo Eun Lee, Young-Hyun Han, Se-In Lee, Do Kyung Kim, Seok-Rae Park, Seong-Lan Yu, Jaeku Kang

**Affiliations:** 1 Priority Research Center, Myunggok Medical Research Institute, College of Medicine, Konyang University, Daejeon, 35365 Republic of Korea; 2 Department of Anatomy, College of Medicine, Konyang University, Daejeon, 35365 Republic of Korea; 3 Department of Microbiology, College of Medicine, Konyang University, Daejeon, 35365 Republic of Korea; 4 Department of Pharmacology, College of Medicine, Konyang University, Daejeon, 35365 Republic of Korea

**Keywords:** Decursinol, Adhesion molecule, Integrin, Implantation, Endometrial receptivity, Exosomes

## Abstract

**Background:**

Embryo implantation is essential for a successful pregnancy, and an elaborate synchronization between the receptive endometrium and trophoblast is required to achieve this implantation. To increase ‘endometrial receptivity’, the endometrium undergoes transformation processes including responses of adhesion molecules and cellular and molecular cell to cell communication. Many natural substances from traditional herbs have been studied to aid in the achievement of successful implantation. In this study, we investigated positive effects on embryonic implantation with decursinol that is a major compound extracted from *Angelica gigas* Nakai known to be associated with promotion of healthy pregnancy in the traditional Korean herbal medicine.

**Methods:**

Expression of cell adhesion molecules after treatment of endometrial epithelial cells by decursinol (40 or 80 μM) was determined using quantitative reverse transcription-polymerase chain reaction (qRT-PCR) and western blot analysis. The alteration of endometrial receptivity by decursinol (40 or 80 μM) was identified with the in vitro implantation model between Ishikawa cells and JAr cell spheroids (diameter, 143 ± 16 μm). Exosomes secreted from Ishikawa cells after treatment of 80 μM decursinol or dimethyl sulfoxide (DMSO) as the vehicle were investigated with invasion of JAr cells and attachment of JAr spheroids to Ishikawa cells.

**Results:**

Decursinol significantly (*P* < 0.05) increased the expression of important endometrial adhesion molecules such as integrin β1, β3, β5 and L-selectin mRNAs and integrin β5 and L-selectin in protein. The adhesion rate of JAr spheroids to decursinol-treated Ishikawa cells also increased significantly which was 2.4-fold higher than that of the control (*P* < 0.05). Furthermore, decursinol induced an increase in the release of exosomes from Ishikawa cells and decursinol-induced exosomes showed autocrine (to Ishikawa cells) and paracrine (to JAr cells) positive effects on our implantation model.

**Conclusion:**

These results propose that decursinol could serve as a new and alternative solution for patients who are infertile.

## Background

Implantation, a process whereby blastocysts attach to and invade the uterus endometrium, is an important gateway for a successful pregnancy. For a successful implantation, elaborate interaction between the developed trophoblast and the receptive endometrium is required [1]. The period termed ‘window of implantation’, which is the phase around day 9 after ovulation, only occurs when the uterus is ready to receive the conceptus [2, 3]. Under regulation by estrogen (E2) and progesterone (P4), the endometrium undergoes numerous transformation processes, including responses of adhesion molecules, cellular and molecular cell-cell communication, extracellular matrix (ECM) remodeling, and expression of many growth factors, cytokines, and their mediators [4]. Integrin, one of the adhesion molecules, is considered to be important as a factor that regulates the establishment of uterine receptivity in general [5].

Failure to establish endometrial receptivity is a major cause of recurrent implantation failure in women [6, 7]. To resolve this problem, assisted reproductive technologies (ART) have been steadily developed [8, 9]. Many previous studies have sought substances such as calcitonin [10], low-dose aspirin [11], GnRH analogues [12] and natural substances extracted from *Paeonia lactiflora* [13], *Perilla frutescens* [14], *Cyperus rotundus* L. [15], *Scutellaria baicalensis* [16] that can help to increase endometrial receptivity. Since traditional herbal remedies have been proven to be safe when administered orally, many researchers have focused on natural substances [13–17]; however, failure of implantation still remains the main challenge in the success of ART [8, 9].

For thousands of years, the root of *Angelica gigas* Nakai (also known as Cham Dang Gui in Korea) has been used as a traditional Korean herbal medicine [18]. *A. gigas* is known to elicit various pharmacological effects including anti-amnestic [19], platelet anti-aggregatory [20], anti-cancer [21–23], anti-inflammation [24], and antibacterial [18] activity. Additionally, it has already been proven to be highly safe with no chronic, hereditary, reproductive, or developmental toxicity when administered orally [25–27]. *A. gigas* is often referred to as the “female ginseng” [28] because of its extensive use to treat gynecological diseases such as dysmenorrhea, amenorrhea, menopausal syndromes, anemia, abdominal pain, injuries, migraine headaches, and arthritis [29–31]. Although *A. gigas* has traditionally been widely used for healthy pregnancy and easy delivery in China, Japan and Korea [31], due to the lack of suitable characterization, it remains unknown whether it has a positive effect on embryonic implantation. The major active compounds of *A. gigas* are essential oils (α-pinene, limonene, β-eudesmol, and elemol) and coumarins (decursinol, decursin, decursinol angelate, demethylsuberosin and nodakenindecursin) [32]. Coumarins extracted from plants have estrogenic activity since they show estrogen receptor relative binding affinities [33–35]. We performed a pre-screening test (trophoblastic JAr cell adhesion) for coumarins derived from *A. gigas* and found that compared to decursin and decursinol angelate, decursinol was the compound most likely to increase the adhesion of trophoblast to the endometrium as it exhibits estrogen response element (ERE) activity [36]. Therefore, in this study, we aimed to determine the effect of decursinol on endometrial receptivity by assessing its effect on the adhesion phase of implantation. We also explored the mechanism by which it enhances endometrial receptivity.

## Methods

### Material

**Fig. 1:**
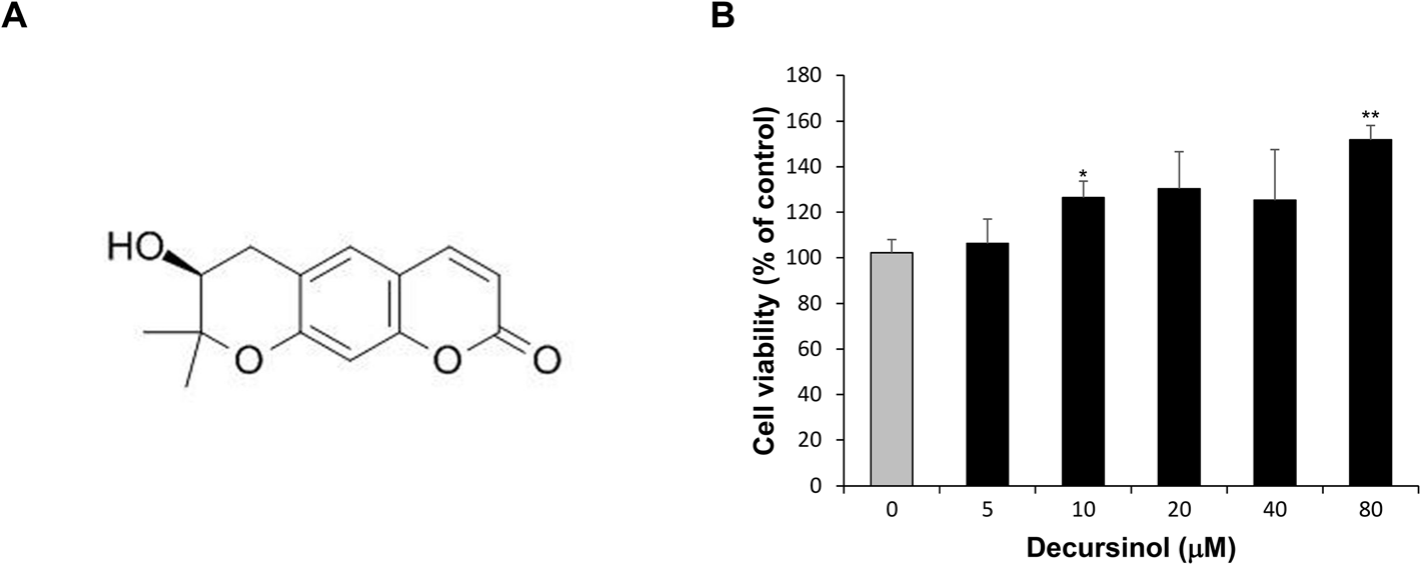
Characterization of decursinol. **a** Molecular structure of decursinol. **b** Cytotoxic effect of decursinol on Ishikawa cells at the indicated concentrations for 24 h. Values are expressed as mean ± SD. The experiment was performed in quadruplicate well. *P < 0.05 and **P < 0.01 are considered significant

Decursinol, the single compound extracted from *Angelica gigas* Nakai was purchased from NPBANK of National Development Institute of Korean Medicine (Gyungsan, Korea); its molecular structure is shown in Fig. 1a. By performing HPLC chromatography, its purity was determined to be over 98.9%.

### Cell lines and cell culture

The human endometrial Ishikawa cell line derived from a human adenocarcinoma was kindly provided by Dr. Jacques Simard (CHUL Research Center, Quebec, Canada). Human choriocarcinoma JAr cells were obtained from the Korea Cell Line Bank (Seoul, Korea). Ishikawa cells were cultured in Dulbecco’s modified Eagle’s medium (DMEM; GE Healthcare, UK) with 10% heat-inactivated fetal bovine serum (FBS; Gibco, Canada) and 1% penicillin / streptomycin. JAr cells were cultured in RPMI-1640 (GE Healthcare, UK) with 10% heat-inactivated FBS and 1% penicillin/streptomycin. Both cell lines were maintained as monolayers at 37 °C in an atmosphere containing 5% CO_2_/air.

### Cell viability assay

The cytotoxicity of decursinol was determined using an EZ-Cytox cell viability assay kit (Daeil Lab Service, Korea). Ishikawa cells were seeded in quadruplicate on a 48-well cell culture plate at 8.0 × 10^4^ cells/well density in a final volume of 0.5 mL/well DMEM medium with 10% FBS and incubated in a humidified 5% CO_2_ incubator at 37 °C for 24 h. Each cell was treated with the indicated concentration of decursinol (from 5 μM to 80 μM) incubated for 24 h, followed by the addition of 50 μL/well of EZ-Cytox reagent to both treated and control wells (without decursinol). After a 1 h incubation, the absorbance at 450 nm was measured with a microplate reader (Molecular Devices, California, USA). Corresponding blanks for each concentration were then used to validate the absorbance. The percentage of absorbance of treated cells was calculated against untreated cells.

### Total RNA isolation and real-time qPCR

Ishikawa cells (8.0 × 10^5^ cells/well) were seeded into each 6-well cell culture plate in DMEM containing 10% FBS. After a 24 h incubation, the culture fluid was replaced with serum-free medium and the cells treated with decursinol (40 or 80 μM) or DMSO as vehicle. After 24 h, the culture fluid was removed and TRIzol reagent (Invitrogen, Thermo Fisher Scientific, Czech Republic) was added directly to each well. Total RNA was extracted according to the manufacturer’s instruction and an equivalent amount of total RNA (2 μg) from each sample reverse-transcribed using dNTP, oligo-dT primer and M-MLV reverse transcriptase (Promega, Madison, WI, USA). Real-time qPCR was performed on a real-time PCR detection system (Bio-Rad, CA, USA) using IQ SYBR green chemistry (Bio-Rad). The amplification conditions for select genes (ITGB1, ITGB3, ITGB5, L-selectin, LIF, E-cadherin and β-actin) were: initial denaturation at 95 °C for 30 s, specific annealing temperature for 15 s, and extension at 72 °C for 15 s for 50 cycles. The homogeneity of the PCR amplification products was verified using the melting curve method. The sequences of the primers and the annealing temperature are given in Table 1. Expression levels of selected genes were quantified following normalization to the expression of β-actin. The results were calculated by the 2^-∆∆CT^ method and fold change evaluated by comparison to the vehicle control. Each experiment was conducted at least in triplicate.

**Table 1:** PCR conditions, amplicon size for each target gene, and primers used in this study

Gene	Primer sequences	Annealing temperature	Size (bp)
ITGB1	Forward: 5′-GTCGTGTGTGTGAGTGCAAC-3′ Reverse: 5′-GCTGGGGTAATTTGTCCCGA-3’	60 °C	318
ITGB3	Forward: 5’-CTGCCGTGACGAGATTGAGT-3′ Reverse: 5′-TGCCCCGGTACGTGATATTG-3’	60 °C	383
ITGB5	Forward: 5’-ACCTGGAACAACGGTGGAGA-3′ Reverse: 5′-AAAAGATGCCGTGTCCCCAA-3’	60 °C	217
L-selectin	Forward: 5’-ATTTCCTGGCACATCATG-3′ Reverse: 5′-ATTGTCTCGGCAGAATCT-3’	56 °C	95
LIF	Forward: 5’-ACAGAGCCTTTGCGTGAAAC-3′ Reverse: 5′-TGGTCCACACCAGCAGATAA-3’	56 °C	78
E-cadherin	Forward: 5’-GGCCTGAAGTGACTCGTAACG-3′ Reverse: 5′-TCAGACTAGCAGCTTCGGAACC-3’	60 °C	201
β-actin	Forward: 5’-CAAGAGATGGCCACGGCTGCT-3′ Reverse: 5′-TCCTTCTGCATCCTGTCGGCA-3’	56 or 60 °C	275

### Western blot analysis

Ishikawa cells (8.0 × 10^5^ cells/well) were seeded into each 6-well cell culture plate in DMEM containing 10% FBS. After a 24 h incubation, the culture fluid was replaced with serum-free medium and cells were treated with decursinol (40 or 80 μM) or DMSO as vehicle. After 24 h of treatment, cells were washed with PBS and collected using Radio-immunoprecipitation (RIPA) solution. Protein quantification was performed by a BCA assay (Thermo Fisher Scientific, Czech Republic). Equal amount of total protein (50 μg/lane) was prepared and separated by sodium dodecyl sulfate-polyacrylamide gel electrophoresis (SDS-PAGE). The proteins were then transferred onto polyvinylidene difluoride (PVDF) membrane and blocked with 5% skim milk in 1 × TBST for 1 h at room temperature. Membranes were incubated with primary antibodies overnight at 4 °C. Primary antibodies of integrin β1 (cat. no. ab52971), β5 (cat. no. ab184312), LIF (cat. no. ab113262), E-cadherin (cat. no. ab76055), CD9 (cat. no. ab2215), CD63 (cat. no. ab59479) were purchased from Abcam (Cambridge, UK); integrin β3 (cat. no. PAB262Hu01) and L-selectin (cat. no. PAA086Hu01) from Cloud-Clone Corp. (Texas, USA); and GAPDH (cat. no. #5174) from Cell Signaling Technology (MA, USA). After the reaction with appropriate secondary antibodies, all signals, except integrin β1, were visualized using ECL chemiluminescence system (SuperSignal™ West Pico PLUS Chemiluminescent Substrate, cat. no. 34577, Thermo Fisher Scientific, Czech Republic). Anti-integrin β1 was visualized using ECL Select reagent (cat. no. #RPN2235, GE Healthcare, UK). Western blot images were quantified by ImageJ software (http://imagej.nih.gov/ij/). Each experiment was performed at least in triplicate.

### Exosome isolation

Ishikawa cells (4.0 × 10^5^ cells) were seeded into each 100-mm dish, in DMEM containing 10% FBS. After a 24 h incubation, the culture media were changed to DMEM containing 1% Bovine serum albumin (BSA; Sigma-Aldrich, Germany) and cells were treated with 80 μM decursinol or DMSO as the vehicle. Twenty-four hours later, each culture medium was collected and centrifuged at 3000 g for 15 min to remove cells and cell debris. Supernatants were transferred to a sterile vessel. To isolate exosomes, ExoQuick™ exosome precipitation solution (SBI System Biosciences, Inc., Mountain View, CA, USA) was added to the supernatants and the mixtures were incubated at 4 °C overnight. The mixtures were centrifuged at 1500 g for 30 min. The pellets obtained were re-centrifuged (1500 g for 5 min) and suspended in 80 μL sterilized PBS. These isolated exosomes were quantified using EXOCET exosome quantitation kit (SBI System Biosciences, Inc., Mountain View, CA, USA) according to the protocol. The particle concentrations were 1.00 × 10^8^ particles/μL in exosomes isolated from decursinol-treated Ishikawa cells (D-Exo) and 4.96 × 10^7^ particles/μL in vehicle control (V-Exo).

### Cell adhesion assay

Ishikawa cells (1.5 × 10^5^ cells/well) were seeded into 24-well cell culture plates with DMEM containing 10% FBS and cultured for 24 h. Medium was then replaced and the cells were incubated in serum-free DMEM containing decursinol (40 or 80 μM) or DMSO for 24 h. JAr spheroids were made using a 96-well cell-repellent surface microplate (Greiner bio-one, Austria) according to the manufacturer’s manual. JAr single cell suspensions (200 cells/100 μL RPMI-1640 medium containing 10% FBS and penicillin/streptomycin) were obtained by trypsinization of monolayer-cultured JAr cells and delivered into each well of the 96 well microplate prior to incubation in a humidified 5% CO_2_ incubator at 37 °C for 24 h. JAr spheroids (20 spheroids/well) were then delivered onto a monolayer of Ishikawa cells in JAr growth medium. After incubation for 1 h at 37 °C, each well was washed with Dulbecco’s Phosphate-Buffered Saline (DPBS; Thermo Fisher Scientific, Czech Republic) once, followed by the addition of JAr growth medium.

D-Exo and V-Exo were added to Ishikawa cells in serum-free DMEM. The JAr spheroids attachment was then examined in the same manner.

The attached JAr spheroids were visualized using a stereomicroscope (SZ 61, Olympus Corp, USA) and counted. Spheroids attachment is expressed as a percentage of seeded spheroids.

### Scanning electron microscope

Pellets containing extracellular vesicles isolated from Ishikawa cells (i.e., exosomes) were vortexed and resuspended in 1 mL of distilled water. The solution was further purified using dialysis tubing cellulose membrane (molecular weight cut-off = 14,000) against 1 L × 3 distilled water for 24 h. A 20 μL solution was then placed on the center of the scanning electron microscope (SEM) sample holder (ϕ = 15 mm) and dried in a ventilation hood for 30 min. A 20 μL volume of ethanol was then directly placed on the dried sample to dehydrate the exosomes. After evaporating the ethanol at room temperature for 30 min, the sample holder was placed in a vacuum chamber for 2 min to eliminate any outgassing from the exosomes and water. To generate a conductive surface, a 10-nm gold coating was applied by sputtering (ion sputter coater) prior to imaging by SEM (SNE-4500 M, South Korea). SEM was used at 10.0 kV and the images obtained were assessed using the Image J software (http://imagej.nih.gov/ij/) software for analysis of exosome size.

### Invasion assay

JAr cell invasion assay was performed by measuring the invasion of cells through Matrigel (BD Bioscience, San Jose, CA) in a 48-well micro chemotaxis chamber (Neuroprobe, Gaithersburg, USA). For the invasion assay, a single 25 × 80 mm piece of Polycarbonate track-etch (PCTE) filter membrane with a 12-μm pore size was coated with a final concentration of 10 μg/mL of Matrigel in serum-free RPMI 1640. Lower wells of the chamber were filled with 30 μL of medium containing 10% FBS which served as the chemoattractant. The chemotaxis chamber was assembled by placing the filter membrane between the lower and upper chamber with the Matrigel-coated side up. A suspension of 5.0 × 10^5^ cells/mL was treated with 2 μL of isolated exosomes per mL with 50 μL placed in each well of the upper chamber. After incubation for 11 h at 37 °C in a 5% CO_2_ incubator, filters were removed, fixed, and stained with Diff-Quik reagents (Sysmex Corporation, Kobe, Japan). Before staining and counting of migrated cells could be performed, non-migrated cells were removed from the top side of the filter. The number of JAr cells that invaded into the lower surface of the filters was counted in 3 random fields under a light microscope at × 200 magnification.

### Statistical analysis

For parametric data, comparisons between groups were performed using analysis of variance (ANOVA) and statistically significant differences were calculated by student t-test, using R program and GraphPad Prism (GraphPad Software, USA). The minimum significance level was a *P* value of 0.05. All experiments were independently performed at least in triplicate.

## Results

### Cytotoxicity of decursinol

A WST-1-based cell viability assay was used to examine the toxicity of decursinol on Ishikawa cell at various concentrations. Although decursinol did not exhibit any cytotoxic effect on Ishikawa cells (Fig. 1b), it displayed a slight proliferative effect at high concentrations. Therefore, we selected the concentrations, 40 μM and 80 μM, for the experimental group.

### Effects of decursinol on the expression of endometrial receptivity markers

**Fig. 2:**
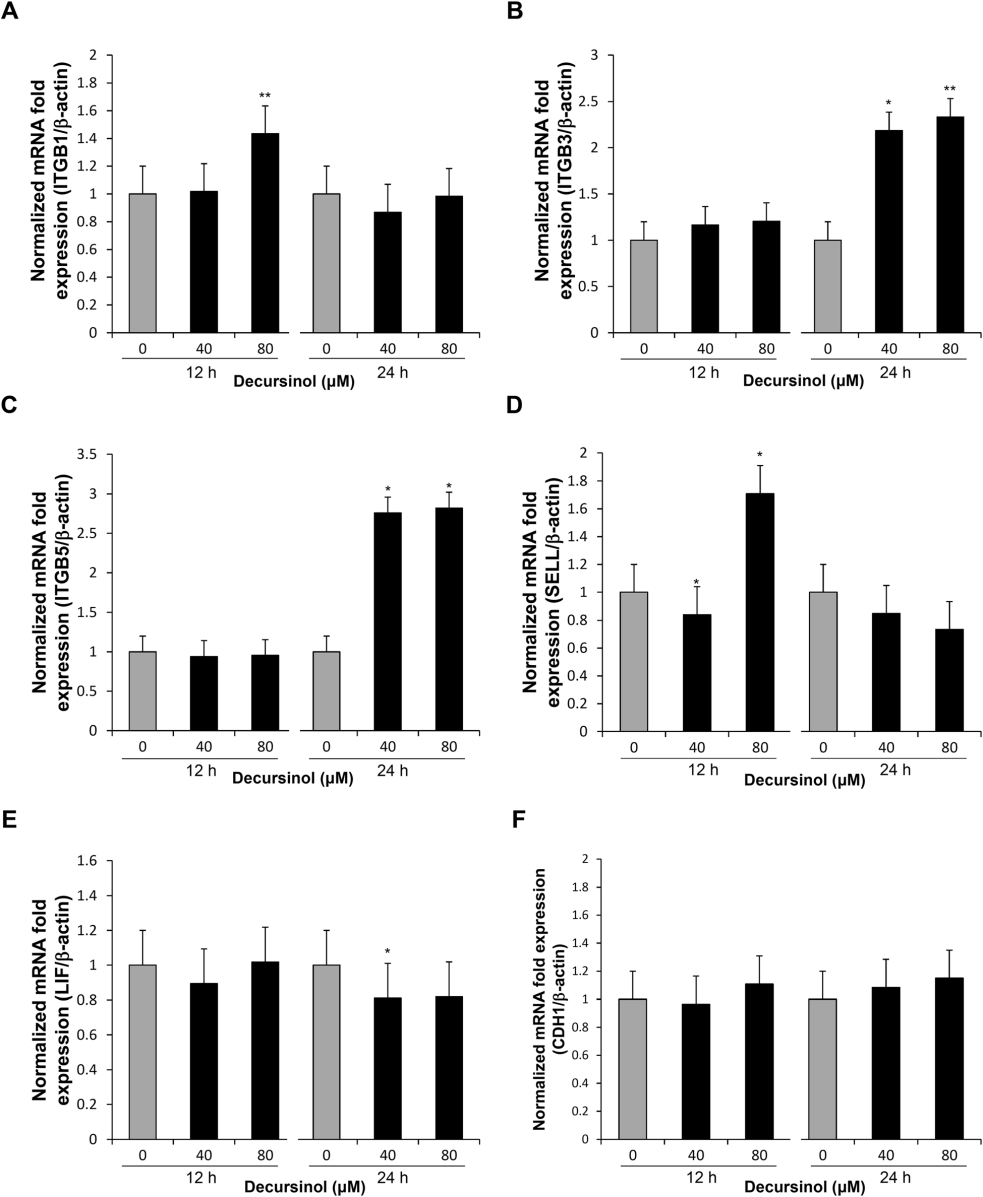
Real-time qPCR was performed to assess the effect of decursinol on the mRNA expression of ITGB1, ITGB3, ITGB5, L-selectin, LIF, and E-cadherin. **a** ITGB1, **b** ITGB3, **c** ITGB5, **d** L-selectin, **e** LIF, and **f** E-cadherin mRNA in the Ishikawa cells transfected at 12 h and 24 h following decursinol treatment (40 or 80 μM). Data shown are normalized mRNA fold change. Values are expressed as mean ± SD. The experiments were repeated in triplicate wells. *P < 0.05 and **P < 0.01 are considered significant

**Fig. 3:**
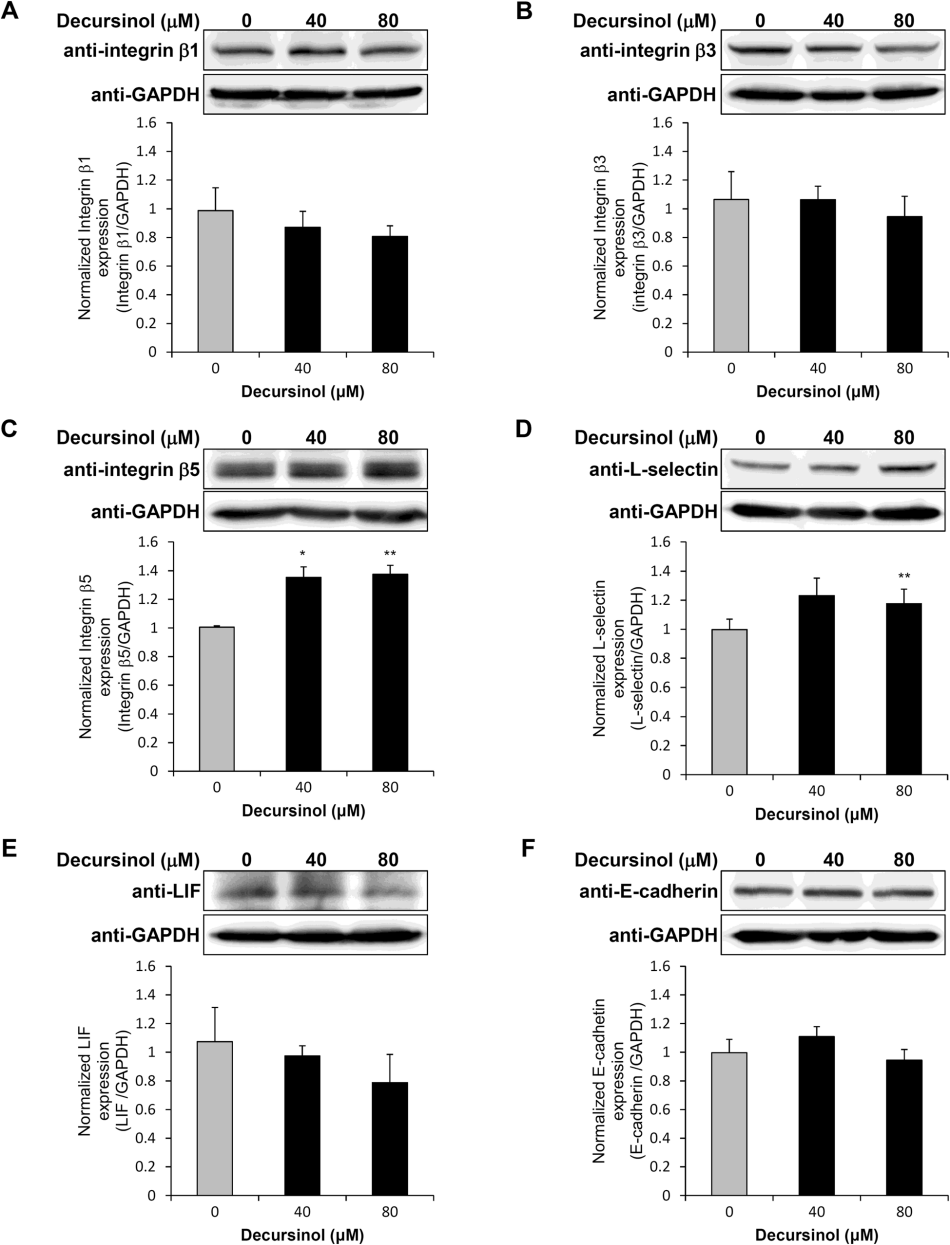
Western blot analysis was performed to assess the effect of decursinol on the protein expression of integrin β1, integrin β3, integrin β5, L-selectin, LIF, and E-cadherin. **a** Integrin β1, **b** integrin β3, **c** integrin β5, **d** L-selectin, **e** LIF, and **f** E-cadherin expression on the Ishikawa cells were measured after 24 h of decursinol treatment (40 or 80 μM). Data are shown as normalized protein expression. Values are expressed as mean ± SD. The experiments were repeated in quadruplicate wells. *P < 0.05 and **P < 0.01 are considered significant

Integrins, L-selectin, and E-cadherin are known as adhesion molecules expressed in human endometrial epithelial cells. LIF is also known as an endometrial receptivity marker that increases the expression of integrin β3 and β5 [11, 37]. To assess the effect of decursinol on the expression of these receptivity markers in endometrial epithelial cells, Ishikawa cells were treated with different concentrations of decursinol (40 μM and 80 μM) and the mRNA expression of receptivity markers measured via real-time qPCR. Integrin β1 and L-selectin were increased significantly when cells were treated with 80 μM decursinol for 12 h (Fig. 2a, d). Similarly, integrin β3 and integrin β5 mRNA levels were significantly increased (greater than two folds) after treatment with 40 and 80 μM decursinol at 24 h (Fig. 2b, c). Although integrin β3 and integrin β5 showed increased expression, LIF level was significantly decreased only at 24 h after treatment with 40 μM decursinol (Fig. 2e). E-cadherin expression showed no significant change at all concentrations and times (Fig. 2f). Protein expression were determined by a western blot analysis after decursinol treatment for 24 h (Fig. 3). The expression of integrin β5 was significantly increased with 40 and 80 μM decursinol treatment (Fig. 3c), while L-selectin expression showed significant increase with 80 μM decursinol treatment (Fig. 3d).

### Effects of decursinol on the attachment of JAr spheroid to Ishikawa cells

**Fig. 4:**
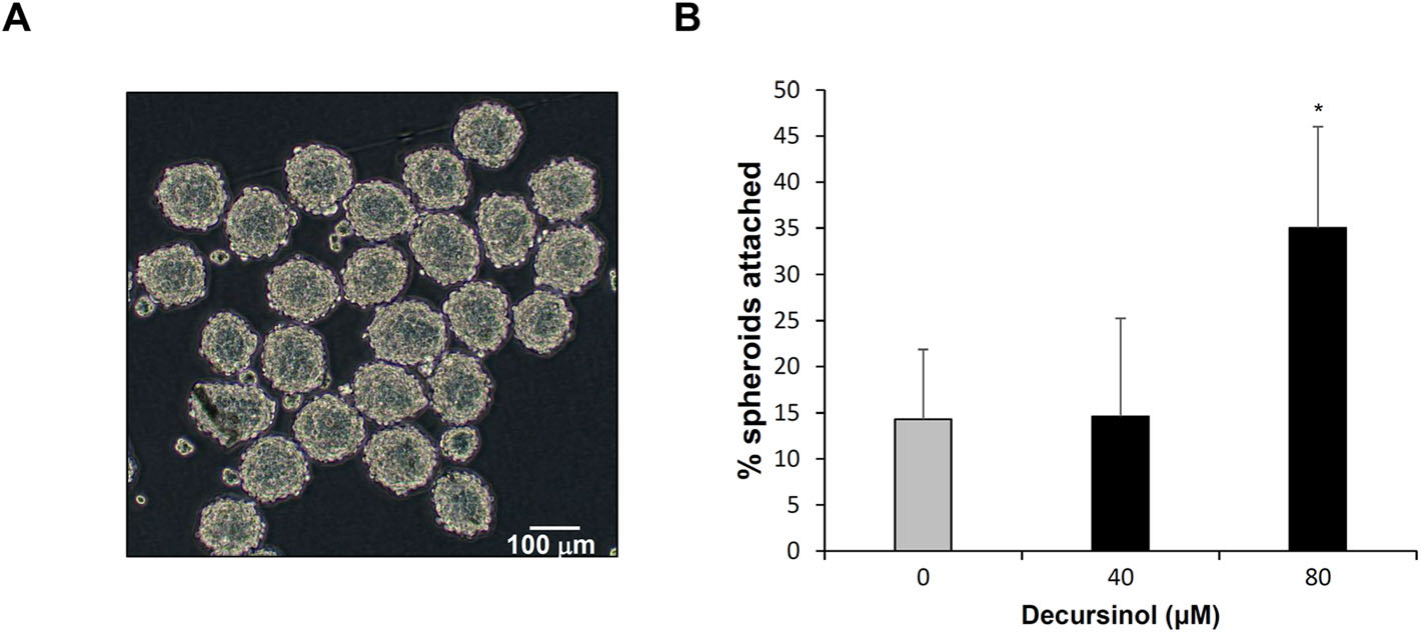
Assessment of the decursinol concentration-dependent attachment rate of JAr spheroids to Ishikawa monolayers. **a** Representative image of cultured JAr spheroids taken using a stereo-microscope at magnification × 100. **b** Attached JAr spheroids were manually counted and the attachment rate was expressed as mean ± SD. The experiment was repeated in quadruplicate wells. *P < 0.05 is considered significant

Fig. 4a shows stereomicroscopic image of JAr spheroids formed with uniform shape. Diameters of JAr spheroids were similar to those of embryos at the period of implantation, with an average of 143 ± 16 μm.

Decursinol significantly increased the attachment rate of JAr spheroids when treated at 80 μM concentration (Fig. 4b). Adhesion rate was 35.0%, a value 2.4-fold higher than that for the control. There was no significant difference (*P* > 0.05) at the 40 μM concentration.

### D-Exo enhances trophoblastic JAr cell invasion

**Fig. 5:**
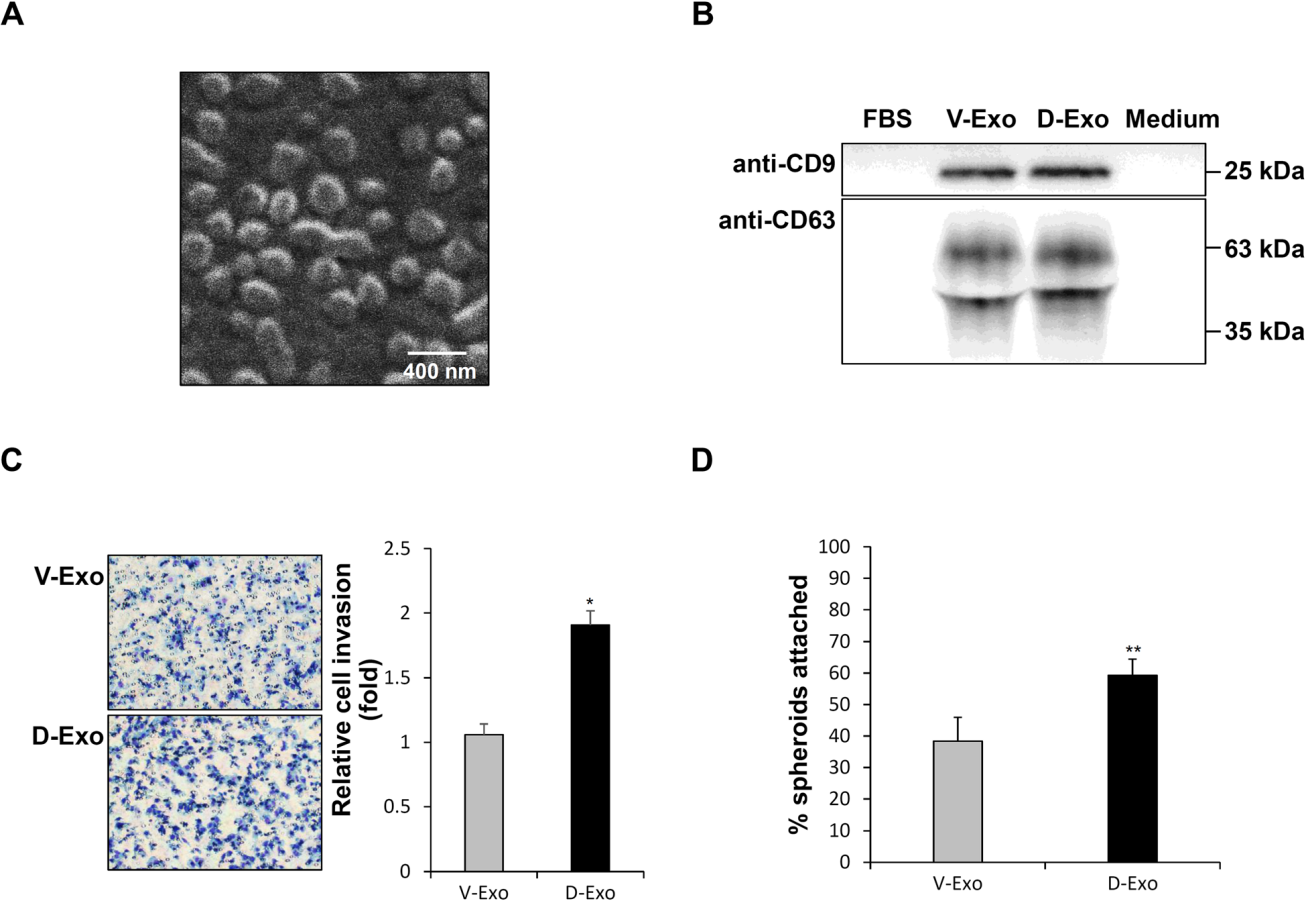
In vitro application and functional effects of D-Exo on implantation model. **a** Scanning electron microscope of exosomes from Ishikawa cells at magnification × 30, 000. **b** Results of western blot analysis for the exosome surface marker, CD9 and CD63, in FBS, V-Exo, D-Exo, and culture medium. **c** JAr cell invasion assay. D-Exo and V-Exo (control) were respectively treated with 2 μL at 5.0 × 10^5^ cells/mL for 11 h. Photomicrographs on the left represent the cells that have passed through Matrigel. Original magnification × 200. The graph on the right shows relative cell invasion rate compared to the control. **d** Effects of D-Exo in the Ishikawa cells on adhesion of JAr spheroids. Data are expressed as mean ± SD. The experiments were repeated in triplicate wells. *P < 0.05 and **P < 0.01 are considered significant. D-Exo, exosomes isolated from decursinol-treated Ishikawa cells; V-Exo, exosomes isolated from vehicle-treated Ishikawa cells

Exosomes were isolated from decursinol-treated Ishikawa cells to confirm their positive role in JAr-Ishikawa cell communication in implantation. Fig. 5a shows a SEM image of exosomes from Ishikawa cells. All exosomes had a spherical shape and an average size of 192.79 ± 21.51 nm. CD9 and CD63 are representative exosomal surface markers and western blot analysis confirmed the presence of exosomes in V-Exo and D-Exo (Fig. 5b). Based on prior quantification of the exosome particles, D-Exo appeared to contain more exosomes than V-Exo. In contrast, the marker proteins were not detected in FBS and culture medium (Fig. 5b). To determine whether D-Exo exhibits positive effects on JAr cells, an invasion assay was performed. The invasion rate of JAr cells increased significantly after treatment with D-Exo compared to V-Exo (Fig. 5c).

### D-Exo increases the rate of attachment of JAr spheroids to Ishikawa cells

To determine the autocrine effect of D-Exo, a JAr spheroid adhesion assay was performed. The rate of attachment of JAr spheroids to V-Exo and D-Exo-treated Ishikawa cells was 38.3 and 59.3% respectively; these values were 1.5-fold greater than that for the control (Fig. 5d). This result demonstrates that not only decursinol itself, but decursinol-induced exosomes also significantly increased the attachment rate of JAr spheroids.

## Discussion

In this study, we determined the effect of decursinol on endometrial receptivity by assessing its effect on the adhesion phase of implantation. We also explored the mechanism by which it enhances endometrial receptivity. We found that decursinol increased the mRNA expression of integrin β1, β3, β5 and L-selectin (Fig. 2), the protein expression of integrin β5 and L-selectin (Fig. 3), and the endometrial adhesion of JAr spheroids (Fig. 4).

Implantation has three steps (apposition, adhesion, and invasion) with each involving complex signaling cascades that are essential for pregnancy. Molecular mediators of adhesion appear to have different action mechanisms or timing [38], and in our study, decursinol appeared to exhibit a different effect for each expression of the adhesion molecules (Figs. 2, 3). Although an increase in marker expression led to an increase in trophoblast attachment rate [39] (Fig. 4), further studies are required to determine the kinetic action mechanism of decursinol, which is the future direction of our research.

Although a study reported that the expression of integrin β3 and β5 was increases in an LIF-dependent manner [39], decursinol did not reveal an apparent LIF-dependent mechanism underlying the increase in integrin β3 and β5 expression in our study; relative to integrin expression, there was no increase in LIF expression (Figs. 2, 3). Our results also showed that the estrogen receptor inhibitor, ICI 182780, did not have an inhibitory effect on decursinol (data not shown), implying that decursinol might not be related to estrogen response element (ERE). Studies are however needed to determine whether the effect of decursinol on implantation is related to glucocorticoid receptors (GRs). Uterine GR signaling as a transcriptional regulator has been deemed important in regulating the immune system and inflammatory response [40] and pre-implantation anti-inflammatory therapies have been reported to increase pregnancy rates [41]. Since decursinol is well known for its anti-inflammatory effect as a coumarin extract [42], further investigations are required to determine whether its positive role in implantation is due to its anti-inflammatory effects via GRs.

Glycosylation is an important post-translational modification of adhesion proteins involved in reproduction, especially in the implantation process [14, 43]. Certain changes in glycosylation of the uterine endometrial epithelial cells are necessary for successful embryonic adhesion to the endometrium [43, 44]. From our western blot results (Fig. 3), the observed band size of proteins such as integrin β1, β3, and L-selectin was found to be slightly higher than the predicted band size; therefore, decursinol is expected to have a glycosylation effect on adhesion proteins in the adhesion phase of implantation. Further studies investigating the function of and glycosylation patterns induced by decursinol will be needed to provide meaningful insights into the actual biological mechanism.

Exosomes released from endometrial epithelial cells play an important role in endometrial-blastocyst interactions during embryo implantation [6]. Exosomes found in endometrial fluid and follicular fluid of ovarian follicles contain microRNAs and exosome- and cell-type-specific proteins. Therefore, these exosomes can mediate cell communication within the blastocyst and endometrium and regulate trophoblast maturation [45–47]. Accordingly, we were interested in the effect of exosomes in the adhesion phase of implantation and thus, sought to explore this molecular aspect. Decursinol-induced exosomes displayed positive effects on both recipient cells (JAr cells) and parent cells (Ishikawa cells) (Fig. 5c, d). Prior studies reported that exosomes can only exhibit positive effects on recipient cells, and not on parent cells (same cell type that the exosome was isolated from) [48]. However, our study paves the way for investigation on a possible autocrine effect being exhibited by decursinol. This is because the exosomes obtained from decursinol-treated Ishikawa cells can affect the same cells in their surroundings. This finding is meaningful since decursinol can induce a positive feedback, leading to a stepwise effect of progressively increasing endometrial receptivity. Decursinol-treated Ishikawa cells released 1.00 × 10^8^ particles/μL, which is about 100% increase compared to the control (4.96 × 10^7^ particles/μL). As shown in Fig. 1b, decursinol has a proliferative effect on Ishikawa cells, increasing the number of Ishikawa cells up to about 50%. Therefore, we assume that among 100% increase of exosome amount, about 50% was due to increase in number of Ishikawa cells, and the other about 50% was solely due to direct effect of decursinol on secreting ability of Ishikawa cells. Which means that direct effect of decursinol on exosome secretion resulted in 2.50 × 10^7^ particles/μL increase of exosomes. Assessing whether decursinol also has an effect on changing the content of exosomes will be our next focus. In addition, in order to elucidate the precise action mechanism of exosomes, microarray analysis should be conducted to derive a profile for the contents of the exosomes. Further studies should be conducted by labeling specific microRNAs and proteins based on their abundance in exosomes to determine their specific functions.

To determine whether implantation rate changes in response to decursinol, an additional in vivo experiment should be performed using an implantation failure mouse model [14, 49]. An in vivo model should also be utilized, with methods similar to those used in our study, for analysis of changes in the expression of endometrial receptivity markers; this would contribute to the possibility of decursinol being employed as a new and alternative therapy for patients who are infertile.

## Conclusion

To summarize, decursinol increased not only the expression of the endometrial adhesion molecules (integrin β5 and L-selectin), but also the rate of adhesion between JAr spheroids and the Ishikawa monolayer and exosome secretion from Ishikawa cells. In addition, these decursinol-induced exosomes had both autocrine (to Ishikawa cells) and paracrine (to JAr cells) positive effects. These results suggest that decursinol could function as a new and alternative therapy by increasing the success rate of implantation in the adhesion phase.

## Data Availability

The raw data for this study is available upon reasonable request to the corresponding author.

## Abbreviations

ART: Assisted reproductive technologies
ECM: Extracellular matrix
ERE: Estrogen response element
GRs: Glucocorticoid receptors
